# Social support in the general population: standardization of the Oslo social support scale (OSSS-3)

**DOI:** 10.1186/s40359-018-0249-9

**Published:** 2018-07-17

**Authors:** Rüya-Daniela Kocalevent, Lorenz Berg, Manfred E. Beutel, Andreas Hinz, Markus Zenger, Martin Härter, Urs Nater, Elmar Brähler

**Affiliations:** 1 Institute and Policlinic for Medical Psychology, University Medical Center Hamburg-Eppendorf, Martinistr, 52, W26, 20246 Hamburg, Germany; 2 Department of General Practice/Primary Care, University Medical Center Hamburg-Eppendorf, Martinistr. 52, W26, 20246 Hamburg, Germany; 3 Department of Psychosomatic Medicine and Psychotherapy, University Medical Center Mainz, Langenbeckstraße 1, 55131 Mainz, Germany; 4 Department of Medical Psychology and Medical Sociology, University of Leipzig, Ph.-Rosenthal-Str. 55, 04103 Leipzig, Germany; 5 Faculty of Applied Human Studies, University of Applied Sciences Magdeburg and Stendal, Stendal, Germany; 6 Department of Psychology, University of Vienna, Vienna, Austria

**Keywords:** Social support, Factor structure, Normative data

## Abstract

**Background:**

The objectives of the study were to generate normative data for the Oslo Social Support Scale (OSSS-3) for different age groups for men and women and to further investigate the factor structure in the general population.

**Methods:**

Nationally representative face-to face household surveys were conducted in Germany in 2008 (*n* = 2524).

**Results:**

Normative data for the Oslo Social Support Scale were generated for men and women (52.3% female) and different age levels (mean age (SD) of 48.9 (18.3) years). Men had mean scores comparable to women (10.1 [SD = 2.3] vs. 10.2 [SD = 2.2]). The EFA resulted in a clear one-factor solution for the OSSS-3.

**Conclusions:**

The normative data provide a framework for the interpretation and comparisons of social support with other populations.

**Electronic supplementary material:**

The online version of this article (10.1186/s40359-018-0249-9) contains supplementary material, which is available to authorized users.

## Background

Social support is a multifaceted and complex construct [1]. Early researchers already pointed out a considerable diversity in the conceptualization, and measurement of social support [2]. Consensus has not been attained, yet. This has led to the application of different measures and conceptualizations in research which makes it difficult to compare findings regarding the role of social support [3].

Generally, social support refers to a psychosocial resource that is accessible in the context of interpersonal contacts, and one’s social network [4]. There is agreement among scholars that the construct can be divided into two sub-categories: structural and functional support [2, 3].

While functional support relates to the experience or expectancy of obtaining support from family, friends and neighbors if required [5], structural support mainly regards the size, and the type of the social network, as well as the frequency of contact within it [3]. According to Cohen and Wills [2], the structural component of social support refers to the existence of relationships, while the functional component assesses the degree to which these relationships serve particular functions and provide resources.

Two components of functional support are often related to mental health in general as well as to depression and depressive symptoms, in particular. These are instrumental, and emotional support [5, 6]. While instrumental support is more tangible in nature, e.g. financial support and practical help [7], emotional support refers to the offer of companionship, and intimacy as well as the provision of caring, and empathy [5, 6].

Some authors further point to the theoretical distinction between actually received support in the past, and perceived availability of support in the future [1]. The latter refers to an individual’s expectancy to obtaining support if required [3–5], while received support refers to the past experience of actually obtaining support [7]. Perceived social support is regarded as a sensitive measure in the context of being able to cope with mental health challenges [4]. Nonetheless, this also holds true for received social support, and in many studies the two concepts are related to each other [1]. This is intuitive and comprehensible because expectations concerning the present, and the future depend on past experiences at least to some degree. Therefore, the two terms will be referred to by the more generic term ‘social support’ in the following.

A final distinction in the conceptualization of social support has to be made for the specificity of the respective instruments. According to Cohen and Wills [2], the generic term of specificity indicates whether an instrument is used to assess specific structures and functions (e.g. marital partners, family members, someone to talk to, financial support) or to assess these structures and functions more globally (e.g. the general social network).

Since consensus in the assessment of social support is still lacking, it is important to validate and standardize theoretically derived instruments. Thus, acceptance can be enhanced, and a more homogeneous use of instruments is encouraged, which in turn leads to a better comparability of results.

Widespread instruments to assess the level of social support are the Duke Social Support Index (DSSI; [8]), Sarason’s Social Support Questionnaire [9] and the Oslo Social Support Scale (OSSS-3; [10, 11]).

The OSSS-3, sometimes referred to as 14-point Oslo Social Support Scale [12], or Oslo 3 Social Support Scale [13], is a brief and economic instrument to assess the level of social support. It can be integrated in larger research projects such as population-based studies without significantly enhancing the effort for both researchers, and participants. The OSSS-3 consists of only three items that ask for the number of close confidants, the sense of concern from other people, and the relationship with neighbors with a focus on the accessibility of practical help.

It has been applied in several large-scale population based surveys in different settings, e.g. the Eurobarometer 58.2 (The European Opinion Research Group, 2003), the third National Survey of Lifestyle, Attitudes and Nutrition in Ireland (SLÁN, 2007), the European KIDSCREEN Study [14], and the Outcome of Depression European Network study [10, 13].

Significant associations with measures of positive mental health [12, 15], mental health problems in adolescents [14], psychological distress and depressive symptoms [12, 16], depression [13], and satisfaction with life [17] have been established in the predicted directions, proving both predictive and construct validity of the OSSS-3. Because of the widespread use of the instrument in different large-scale research settings, and its brevity, the OSSS-3 is considered the measure of choice in the current paper.

Despite the attested feasibility and validity [17, 18], the factor structure of the OSSS-3 has remained unclear up to date. In addition, normative data from the general population or other populations have not been generated, yet. Such data provide a framework for the interpretation and comparisons with other populations.

The objectives of the study were to generate normative data for the Oslo Social Support Scale (OSSS-3) for different age groups for men and women and to further investigate the factor structure in the general population.

## Methods

### Study sample

A nationwide survey, representative of the German general population, was conducted with the assistance of an institute specialized for demographic research (USUMA, Berlin) according to the German law of data protection (§30a BDSG, German law of protection of data privacy) and with written consent and in accordance with the guidelines in the Declaration of Helsinki. The ethics committee of the University of Leipzig approved the study. All adult participants provided their written informed consent to participate in this study. Also, written informed consent from the next of kin, caretakers, or guardians on behalf of the minors/children enrolled in the study was obtained. These consent procedures were approved by the ethics committee. The basic population for the data collection is made up of the German population aged at least 14 years and living in private households in 2008 (*N* = 2524). Age, sex, and educational level were the major criteria for representativeness according to the register of the Federal Elections. Two callbacks had to be without success before an address was considered a failure. The sampling procedure consisted of sample points, household, and persons in the last stage. Target households within the sample points were determined using the random-route procedure: choosing sample point areas within Germany, randomly choosing households within these areas, and randomly choosing target persons within these households.

Within this larger survey, the study participants were interviewed using a structured self-report questionnaire including the following instrument.

### Instrument

The OSSS-3 consists of three items assessing the level of social support. It has been recommended for epidemiological and population-based surveys. Consequently, it can be considered a suited instrument for the current thesis [11, 13]. Items are the following:

**Oslo 1**: How many people are so close to you that you can count on them if you have great personal problems?

1. *‘none’*
2. *‘1–2’*
3. *‘3–5’*
4. *‘5+’*

**Oslo 2**: How much interest and concern do people show in what you do?

1. *‘none’*
2. ‘*little’*
3. *‘uncertain’*
4. *‘some’*
5. ‘*a lot’*

**Oslo 3**: How easy is it to get practical help from neighbors if you should need it?

1. ‘*very difficult’*
2. ‘*difficult’*
3. ‘*possible’*
4. ‘*easy’*
5. ‘*very easy’*

The sum score ranges from 3 to 14, with high values representing strong levels and low values representing poor levels of social support. This continuous score was used to generate the normative data for the OSSS-3 for each scoring point as well as to determine group differences according to age and sex.

Referring to Bøen and colleagues [18], the OSSS-3 sum score can be operationalized into three broad categories of social support.

- 3–8 *poor social support*
- 9–11 *moderate social support*
- 12–14 *strong social support*

### Data analysis

As measure of the test’s reliability Cronbach’s alpha was calculated. The factor structure of the OSSS-3 was tested with exploratory factor analysis (EFA). The common CFA fit indices cannot be properly calculated when there are only three items. A model with only three items is saturated. You have 6 covariance moments (three variances and three covariances), and you are estimating 6 parameters (factor variance, two loadings, three measurement error/unique variances). The problem has an exact solution, and zero objective function means that Amos or Mplus successfully find that solution. It also means that one cannot use the overall goodness of fit test to see how well your model fits. There is simply no further information to extract from the covariance matrix.

The percentiles were calculated according to the following formula: percentile rank = 100* (m + 0.5 k)/N, where m is the number of members of the sample who obtained a score that was lower than the score of interest, k is the number who obtained the score of interest, and N is the overall normative sample size.

Statistical analyses were conducted using SPSS 18 with an α-level of 5% and AMOS 23.

## Results

### Sample characteristics

Characteristics of the study sample closely match those of the total German population [19] and the US National Comorbidity Survey Replication [20] on gender (women: 53.7, 51.7, and 55.5%, respectively), employment status (unemployed: 5.8, 7.1, and 3.9%, respectively), marital status (married: 53.7, 57.2, 57.2%), and educational level. In addition, mean age in our study sample was similar to the mean age in the German general population aged 14 years or older (48.9 vs. 46.9 years) (Table 1).

**Table 1:** Demographic characteristics of the sample

Variables	*n*	%
Sex		
Male	1204	47.7
Female	1320	52.3
Age Groups, years		
14–24	302	12.0
25–34	298	11.8
35–44	453	17.9
45–54	448	17.7
55–64	409	16.2
65–74	406	16.1
≥ 75	208	8.3
Marital Status		
Married/Living together	1313	52.0
Separated/Living Apart	24	1.0
Single	606	24,0
Divorced	262	10.4
Widowed	319	12.6
Net Household Income		
< 1250€	559	23.3
1250 - < 2500€	1243	51.7
> 2500€	602	25.0
Employment		
Yes^a^	2384	94.5
No	140	5.5
Education		
None completed	42	1.7
Pupils	119	4.7
High School	1943	76.9
College	297	11.1
University	141	5.6

^a^(includes part-time employed)

Considering the sample as a whole, the mean score of social support was 10.16 (*SD* = 2.25). The median was 10 and scores ranged from 3 to 14. This represented the whole range of the OSSS-3. The mean score was located in the category of ‘moderate social support’, which was supported by the fact that this was by far the most frequent (45.3%) category (Tables 2 and 3).

**Table 2:** Frequency distribution of social support in the general population

Social Support	*n*	%
Strong (OSSS = 12–14)	766	30.3
Moderate (OSSS = 9–11)	1143	45.3
Poor Support (OSSS = 3–8)	610	24.2

*OSSS* Oslo Social Support Scale

**Table 3:** Social Support – mean scores as a function of sex and age

Variables	*n*	OSSS *M* (*SD*)	Group differences^a^	Effect Size^b^
Sex			*p* > .05	*d* = .03
Male	1201	10.13 (2.29)		
Female	1318	**10.19**^c^ (2.21)		
Age group, yr			*p < .*001	*d* = .39
14–24	302	**10.66** (2.07)		
25–34	297	10.31 (2.29)		
35–44	453	10.41 (2,20)		
45–54	448	10.04 (2.36)		
55–64	407	9.90 (2.18)		
65–74	404	9.98 (2.17)		
≥ 75	208	9.79 (2.40)		

*OSSS* Oslo Social Support Scale

*Notes*: ^a^ Group differences were performed using Kruskal-Wallis- and Mann-Whitney-U-Tests; for each variable, the subgroups for that variable with the highest and lowest mean scores were used for calculation of effect size ^b^ Effect Size as Cohen’s *d*; ^c^ The bolded means represent the subgroups with the highest mean score

### Internal consistency

For the OSSS-3, the internal consistency could be regarded as acceptable with *α* = .640. The relatively low value was probably due to the scale’s brevity. Normally, brief scales have difficulties in reaching high *α* -values because Cronbach’s Alpha strongly depends on the item number, i.e. *α* increases simply as a function of the scale’s length, which is one of the caveats mentioned before [21]. Since the OSSS-3 is a very brief and economic scale, the internal consistency can be considered as satisfying (Table 4).

**Table 4:** Item characteristics of the OSSS

Item	*M* (*SD*)	Discriminatory Power	*α,* if item is deleted
Close Network	2.81 (0.77)	*r*_*it*_ = .403	*α* = .615
Concern/Interest of others	3.72 (1.14)	*r*_*it*_ = .511	*α* = .460
Neighbors	3.63 (1.00)	*r*_*it*_ = .469	*α* = .514
OSSS-3 (total)	10.16 (2.25)		

*Notes*: *α =* Cronbach’s Alpha; r_it_ = discriminatory power of the item

*OSSS* Oslo Social Support Scale

### Construct validity

An EFA was conducted to determine the factor structure of the OSSS-3 on the basis of the correlative associations between its items. The Principal Component Analysis (PCA) was deployed as the method of choice and components were orthogonally rotated, using the varimax-rotation procedure with Kaiser-normalization to obtain uncorrelated factors. Since a necessary prerequisite for a PCA are substantial correlations between the items [22], the respective correlation matrix is presented in Table 5. The correlations between the items were all positive and within the critical threshold between *r* = .30 and *r* = .90 [23]. The mean correlation was *r* = .377. The determinant of the matrix, which is an important diagnostic tool to check for singularity or multicollinearity, was 0.638, which is far beyond the critical limit of 0.000001 [23].

**Table 5:** Correlation-matrix of the OSSS items

Items	Close Network	Concern of others	Neighbours
Close Network			
Concern of others	.375**^a^		
Neighbors	.320**	.463**	

*OSSS* Oslo Social Support Scale

*Notes*^: **^ *p* < .01; ^a^ Spearman’s correlation coefficients between the items

The PCA resulted in a clear one-factor solution for the OSSS-3. The extracted factor had an eigenvalue of 1.756 and explained 58.54% of the total variance. Table 10 presents the factor loadings of the three items, which were all invariably high. Affirmatively, the visual inspection of the scree plot also supported the one-factor solution. There was a clear knee in the plot that was supportive of the results suggested by the eigenvalue criterion. The results of the PCA were important because the one-factor solution justified the interpretation of the OSSS-3 sum score as a global factor of social support (Table 6).

**Table 6:** Factor loadings of the OSSS items

Items	Factor loadings on Component 1	Communalities after extraction
Close Network	.714	.510
Concern and interest of others	.808	.653
Neighbors	.770	.593
Eigenvalue	1.756	
% of variance	58.54	

*OSSS* Oslo Social Support Scale

### Normative data

Table 7 summarizes the normative data for the different age levels and both genders. Percentiles from this table can be used to compare an individual subject’s OSSS-3 score with those determined from the general population reference group based on age and gender.

**Table 7:** Normative values of the OSSS-3

	Total	Men							Women						
	14–91 yr.	14–24	25–34	35–44	45–54	55–64	65–74	> 75	14–24	25–34	35–44	45–54	55–64	65–74	> 75
	*n* = 2524	*n* = 160	*n* = 137	*n* = 202	*n* = 224	*n* = 183	*n* = 204	*n* = 91	*n* = 142	*n* = 160	*n* = 251	*n* = 224	*n* = 224	*n* = 200	*n* = 117
*M*	10.16	10.66	10.18	10.50	9.78	9.85	10.11	9.70	10.66	10.43	10.34	10.31	9.94	9.84	9.85
*(SD)*	2.07	2.07	2.27	2.20	2.55	2.06	2.21	2.56	2.09	2.31	2.20	2.13	2.27	2.12	2.27
Sum score	Percentile^a^														
3	0.3	0.0	0.7	0.0	1.3	0.0	0.0	1.1	0.0	0.6	0.8	0.0	0.0	0.0	0.0
4	0.8	0.6	0.7	0.0	3.1	0.0	0.5	3.3	0.0	1.3	1.2	0.0	0.9	0.0	0.0
5	1.8	1.3	2.2	0.5	4.0	0.5	1.5	6.6	1.4	2.5	1.6	0.9	1.8	2.0	0.9
6	5.3	2.5	5.1	5.4	10.3	2.2	5.9	8.8	4.2	5.0	4.4	3.1	6.3	5.0	7.7
7	15.0	8.8	15.3	12.9	21.9	16.4	16.7	20.9	8.5	13.1	10.4	11.2	19.6	16.0	20.5
8	24.2	15.0	23.4	16.8	31.3	30.6	24.5	31.9	14.8	21.3	20.7	24.1	27.2	27.5	32.5
9	36.2	28.1	35.0	31.7	42.4	42.1	34.8	45.1	28.2	30.0	32.3	34.8	40.2	44.0	39.3
10	52.0	41.9	51.8	46.5	57.1	60.7	51.5	59.3	43.0	45.6	50.2	48.7	54.9	61.5	56.4
11	69.9	61.9	70.1	63.9	72.3	76.5	72.5	73.6	59.2	64.4	69.3	68.3	72.8	74.0	74.4
12	84.0	79.4	84.7	79.7	84.8	88.5	86.3	87.9	81.0	79.4	81.7	83.5	86.6	88.0	85.5
13	94.6	94.4	93.4	92.6	93.3	97.3	94.6	91.2	93.7	93.8	93.6	94.6	95.5	98.0	97.4
14	100.0	100.0	100.0	100.0	100.0	100.0	100.0	100.0	100.0	100.0	100.0	100.0	100.0	100.0	100.0

For example, an OSSS (*OSSS* Oslo Social Support Scale) score of 11 in a 24-year-old man indicates a percentile rank of 69.9% in the total population and of 61.9% in a group of subjects of the same age and gender

## Discussion

There were significant differences in the level of social support between different age groups. As a general trend, it was observable that the higher the age, the lower was the reported level of social support. The youngest age groups in return had the highest levels of support. These results fit well into the literature so far and do not contradict empirical findings by other researchers [4]. Furthermore, the growing body of literature concerning the problem of loneliness and social isolation in the elderly [24, 25], the development of screening scales to assess social isolation in this group [26], as well as the development of interventions to face that problem bolster these results [27]. Some authors refer to the social isolation of elderly people as a major social problem in Western societies [28]. In a longitudinal, population-based study from Finland [29], it was found that loneliness increased with age and that the percentage of people who feel lonely was higher in older age groups, mainly due to an increasing disability and a decreasing social integration. This is consistent with the suggestion by Sonnenberg and colleagues [30] that many swift alterations in the social networks of elderly people can occur due to abrupt changes in health, functional capacity, illness or the death of important others [30].

The consistently lower levels of social support and the problem of social isolation in elderly people in combination with the rapid demographic changes and ageing populations in Western countries might be another cause for concern. Projections of the population development in Germany by the year 2050 assume that the age structure will substantially change. The population will be older and smaller than nowadays [31]. This is mainly due to the combination of low fertility and low mortality. The critical questions in the context of the present research will be, how this population development will influence the level of social support in the affected countries, and which effects a possibly lower, general level of social support in the society will have on diverse mental and physical health issues, one major topic among them being depression and the severity of depressive symptoms.

Men and women did not differ significantly in their levels of social support. Former studies consistently reported a higher level of social support and a stronger emotional attachment to their social networks in women compared to men [4, 13, 30, 32, 33]. These findings could not be replicated in the current study what might reflect the beginning of a general change in the traditional gender roles in the present. Was it relatively common for women to take care for the family, to raise children, and to maintain social relationships, while men were mainly responsible for the family income, these traditional role allocations have begun to change substantially due to political activities as well as simultaneous changes in the respective gender role models and stereotypes. Important keywords that describe this development are gender equality and the feminization of men. Consistently, some empirical evidence for a partly changed meaning of social support comes from Sonnenberg and colleagues [30] who could demonstrate that a lack of emotional support and the lack of a partner in the household were more detrimental for men than for women with regard to depression. A small network size even predicted the onset of depression only in men but not in women.

This was the first study to present normative data for the OSSS-3, a brief and economic instrument for the assessment of the level of social support. These data can be used for several purposes. It is now possible to validly classify an individual’s score on the OSS-3 and compare it either with the reference score in the general population or in his or her respective age group. By doing so, it becomes possible to classify, which level of social support this particular individual has available relative to his or her reference group. For example, an OSSS-3 score of 12 in a 20-year-old man indicates a percentile rank of 79.7% in his reference group (males in the age between 14 and 24) and a percentile rank of 84.0% in the total population. This means that, in his reference group, 79.7% have the same or a lower score and vice versa, only 20.3% have a higher score. The application of percentiles has the advantage to be independent of distribution assumptions as well as an easy interpretability. Furthermore, it is now possible to compare levels of social support between different populations on the basis of these comparative values and to use these data as reference categories in community studies [34, 35].

Concerning the factor structure of the OSSS-3, a one-factor solution fitted the data best as the result of a Principal Component Analysis using the varimax-rotation procedure with Kaiser-normalization to obtain uncorrelated factors. Although some authors already attested the feasibility and validity of the OSSS-3 [17, 18], the factor structure has not been clarified and reported, yet. Next to the provision of normative data, this was another important objective of the current study, to address and overcome that methodological shortcoming. In the light of a very heterogeneous conceptualization and administration of the social support construct [1, 2], it is important to promote theory-based, well-validated and standardized measuring instruments. Social support can be and already has been conceptualized on different levels of specificity (e.g. [5, 13]). This makes it difficult to compare findings regarding the role of social support for different health care issues [3]. The reported one-factor solution supports the idea that social support is an overarching construct that aggregates facets such as structural and instrumental support, and thus can be interpreted on a more generic level. This is an important methodological advancement, which further contributes to the theoretical clarification of the social support construct. The reported psychometric properties of the OSSS-3 support the reliability of the scale. Although it is very short, containing only three items, the internal consistency was satisfying.

In conclusion, complementing the suggestions by Bøen and colleagues [18] as well as Glaesmer and colleagues [16] regarding the validity and feasibility of the OSSS-3 and its already widespread application in large-scale research settings (e.g. [13, 14]), this paper delivered some important, yet missing methodological findings that further support the scale’s reliability, contribute to the structural and factorial clarity, and endorse the applicability in different contexts such as individual classification and intercultural comparisons [13, 14, 17, 18]. Yet, the reliability of the scale remains a question, as e.g. test-retest reliability could not be reported.

Due to these findings, an intensified use of the OSSS-3 in future research projects might be promoted. This would help to overcome the methodological heterogeneity in the conceptualization and assessment of social support and make the results of different studies and research groups better comparable. One apparent advantage of the OSSS-3 is the brevity and economic assessment of social support with only three items. As a result, the scale can be incorporated into larger research projects, such as population-based studies, without significantly enhancing the effort for participants and researchers.

To further validate the OSSS-3, it would be interesting to assess the associations with other measures of social support. In particular, the associations with more extended or longer scales such as the DSSI [8] would be illuminating. If the OSS-3 showed substantial associations with these scales, implying that they measure the same construct, this would be another argument for an intensified administration in future projects, due to the higher economy of the OSSS-3.

Regarding the distribution of social support in the German general population, a majority of 75% reports to have at least moderate levels. One third of the population even reports to have strong levels of support. Nonetheless, there is about one quarter in the general population who report to have only poor levels of support. Since these data are descriptive in nature and no population-based studies from different countries could be identified that deliver values to compare the reported ones with, no further speculations will be undertaken concerning the meaning of these figures. Just a little note of caution shall be sounded at this juncture. Despite the majority reporting moderate and high levels of support, almost 25% of the population feels poorly supported what might be a cause for concern. The future development has to be monitored carefully.

For the purpose of intercultural comparisons with regard to the levels of social support and the valid interpretation of the reported figures, it would be desirable if future population-based studies assessed the general level of social support in different populations and delivered normative data from these cultures. For example, it is worth knowing if there are differences between individualistic, mainly Western and collectivistic, mainly Eastern cultures [36]. These two cultural types differ markedly in the construal of the self and the meaning of the community. Individualistic cultures are shaped more competitively, emphasize the importance of the individual, and have an independent view of the self, while collectivistic cultures are shaped more familial, emphasize group ties and social bonds stronger, and have an interdependent view of the self [37]. Consequently, members of collectivistic cultures emphasize the harmonious interdependence with other people, while members of individualistic cultures strive for independence of others. In consequence, this could lead to a higher level and estimation of social support in collectivistic cultures compared to individualistic cultures.

Furthermore, longitudinal studies should investigate if and how the level of social support is changing over time. This is especially intriguing in the light of rapid demographic changes such as an ageing population and major societal transformations such as an increasing technologization and digitalization. Research questions might concern if the ongoing ageing of the population leads to decreasing levels of support; which effects the rapid spread of social networks has on social relationships and social supports in the “real world”; and what meaning social support has for the generation of the digital natives.

## Conclusions

The normative data provide a framework for the interpretation and comparisons of social support with other populations. Evidence supports reliability and validity of the OSSS-3 as a measure of social determinants of health in the general population.

## Additional files

Additional file 1: Raw data. (XLS 1306 kb)

Additional file 2: Key for raw data file (DOCX 12 kb)
