# Supplementary material for: Social support in the general population: standardization of the Oslo social support scale (OSSS-3)
Source: BMC Psychol. 2018 Jul 17;6:31. doi: 10.1186/s40359-018-0249-9 (PMC6050647; doi:10.1186/s40359-018-0249-9)
Supplement: Supplementary file 2 — Key for raw data file (DOCX 12 kb) [file 40359_2018_249_MOESM2_ESM.docx]

File name: R15_OSSS

Title of data: Oslo Social Support Scale in the general population

Description of data:

Lfd: ID

| z10 | Wie viele Menschen stehen Ihnen so nahe, dass Sie sich auf sie verlassen können, wenn Sie ernsthafte persönliche Problem |
| --- | --- |
| z11 | Wieviel Interesse zeigen andere Menschen für Sie und das was Sie tun? |
| z12 | Wie einfach ist es, von Ihren Nachbarn praktische Hilfe zu bekommen, wenn Sie diese brauchen? |
| sex | Geschlecht |
| s3a | Staatsangehörigkeit |
| alter | Alter |
| s5 | Familienstand |
| s5a | Partnerschaft |
| s6 | Erwerbstätigkeit |
| s7 | Zu welchem Berufskreis gehört der Beruf, den Sie selbst ausüben bzw. zuletzt ausgeübt haben? |
| s8 | Schulabschluss |
| s8a | abgeschlossene Lehre |
| s9 | Religion oder Konfession |
| s15 | Wie viele Personen leben ständig in Ihrem Haushalt ... Sie selbst mit eingeschlossen? |
| s16a | Wie viele davon sind ... Kinder unter 3 Jahren? |
| s16b | Wie viele davon sind ... Kinder von 3 - 5 Jahren? |
| s16c | Wie viele davon sind ... Kinder von 6 - 13 Jahren? |
| s16d | Wie viele davon sind ... Personen von 14 - 17 Jahren? |
| s16e | Wie viele davon sind ... Personen ab 18 Jahren? |
| s16f | Wie viele davon sind ... Personen ab 14 Jahren? |
| s17 | Personen des Haushaltes mit eigenem Einkommen |
| s18a | eigenes Einkommen |
| s18b | Haushaltsnettoeinkommen |
| b_land | Bundesland |
| land | West/ Ost |
| partner | Partnerschaft |
| arblos | arbeitslos |
| beruf | Beruf |
| kirche | Kirchenzugehörigkeit |
| h_einkom | Haushaltseinkommen |
| hauseink |  |
| bildung | Schule/ Studium |
| arbeit | Arbeitsstatus |
| sta_land | Stadt/ Land |
| z11u | Interesse anderer an dem, was Sie tun |
| z12u | prakt.Hilfedurch Nachbarn |
| oss_scal | Oslo Social Support Scale |
| oss_grup | Gruppen OSS |
